# Polyunsaturated fatty acid and oxylipin profiles of *Angiostrongylus cantonensis* during cerebral-to-pulmonary migration illuminate parasite and host microenvironmental lipid signatures

**DOI:** 10.1186/s13071-026-07469-z

**Published:** 2026-06-02

**Authors:** Zhengxu Tang, Jiaying Lai, Yashan Song, Song Li, Lihua Xiao, Yaoyu Feng, Dongjuan Yuan

**Affiliations:** State Key Laboratory of Animal Disease Control and Prevention, Center for Emerging and Zoonotic Diseases, College of Veterinary Medicine, South China Agricultural University, Guangzhou, 510642 China

**Keywords:** *Angiostrongylus cantonensis*, Polyunsaturated fatty acid, Oxylipin, Biosynthesis, Enzyme

## Abstract

**Background:**

Metabolic streamlining as a consequence of parasitism has resulted in the loss of lipid biosynthetic genes in nematodes. *Angiostrongylus cantonensis*, a zoonotic neurotropic parasite that causes eosinophilic meningitis in mammals, completes a complex migration through the rat brain before maturing in the pulmonary arteries. Polyunsaturated fatty acids (PUFAs) and their bioactive metabolites (oxylipins) are known to modulate nematode survival; however, their abundance and endogenous biosynthetic capacity in *A. cantonensis* remain elusive.

**Methods:**

We integrated comparative genomics, stage-specific transcriptomics, and lipidomics to reconstruct the expression patterns of the biosynthetic enzymes in *A. cantonensis* and characterize their dynamic PUFA and oxylipin profiles during the neuro-pulmonary transition from brain-residing fourth-stage (L4) to lung-migrating L5 larvae.

**Results:**

Genomic and transcriptomic analyses revealed a streamlined biosynthetic repertoire in *A. cantonensis*. The worm lacks Δ6 desaturase and canonical cyclooxygenase (COX), lipoxygenase (LOX), and cytochrome P450 (CYP) enzymes, and expresses only residual levels of *ptges2* and *lta4h*, which are markedly lower than those in free-living *Caenorhabditis elegans* across developmental stages. Using liquid chromatography-tandem mass spectrometry, L4–L5 larvae residing in the rat brain maintained stable PUFA and oxylipin profiles. In contrast, L5 larvae migrating to the lungs accumulated *n*-6 PUFAs and anti-inflammatory hydroxy eicosatetraenoic acids, epoxy eicosatrienoic acids and hydroxy docosahexaenoic acids, concurrent with reduced levels of pro-inflammatory prostaglandins and 5-oxo-eicosatetraenoic acid.

**Conclusions:**

These stage- and tissue-specific lipidomic shifts correlate with distinct host microenvironments encountered during migration. These findings provide a foundation for understanding the evolutionary adaptation of lipid metabolism in parasitic nematodes.

**Graphical Abstract:**

**Figure:**
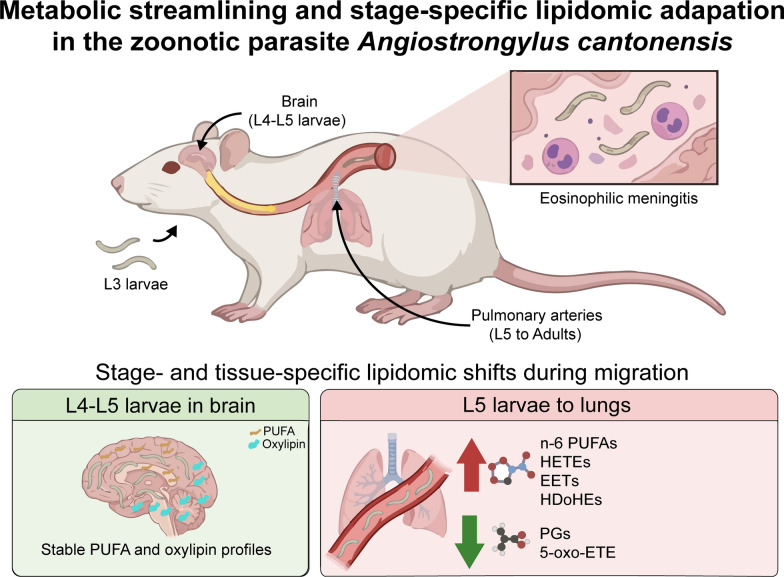

**Supplementary Information:**

The online version contains supplementary material available at 10.1186/s13071-026-07469-z.

## Background

*Angiostrongylus cantonensis* is a food-borne nematode and the causative agent of angiostrongyliasis, a zoonotic disease affecting both humans and animals [1, 2]. Its complex life cycle involves intermediate molluscan hosts, definitive rat hosts, and a variety of non-permissive hosts, including humans, dogs, monkeys, pigs, cows, and mice. In rats, the larvae migrate to the brain, where they undergo two molts to develop into fifth-stage (L5) larvae within approximately 28 days. These L5 larvae then migrate to the pulmonary arteries, where they mature into adults and commence egg laying [2, 3].

Accumulating evidence indicates that polyunsaturated fatty acids (PUFAs) and their bioactive metabolites, oxylipins, function as stage-specific developmental signals, neuromodulators, and potent immunomodulators, thereby promoting parasite survival in immunocompetent hosts [4–9]. In the free-living *Caenorhabditis elegans*, *n*-6 PUFAs act as metabolic rheostats that modulate oocyte lipid/yolk content and reproductive efficiency [8, 10–12], extend the nematode’s lifespan by mitigating oxidative stress [13, 14], and induce ferroptotic neurodegeneration in its sensory circuits [15, 16]. Moreover, PUFAs serve as primary regulators of sensory neurons, enabling the worm to evade thermal, osmotic, oxidative, heavy metal, and hypertonic stress [17, 18]. F-series prostaglandins (PGs) facilitate sperm navigation to the fertilization site in *C. elegans* [7, 19, 20], whereas dihomo-γ-linolenic acid (DGLA)-derived 8,9- and 14,15-epoxyeicosadienoic acids induce germline dysplasia [12]. The arachidonate metabolite 20-hydroxyeicosatetraenoic acid (20-HETE) dampens pharyngeal pumping in *C. elegans* [21]. In parasitic species, live or moribund filariae release prostaglandin E2 to modulate host immunity toward an immunopathological environment favorable for parasite survival [22], and the whipworm *Trichuris suis* secretes prostaglandin E2 to suppress pro-inflammatory responses in human dendritic cells [23]. Thus, these findings indicate that lipid-mediated signaling plays a critical role in nematode fitness and survival across both free-living and parasitic lineages.

PUFA biosynthesis is conserved between mammals and *C. elegans* and is catalyzed by fatty acid desaturases and elongases. In mammals, oxylipin biosynthesis is primarily mediated by cyclooxygenase (COX), lipoxygenase (LOX), and cytochrome P450 (CYP) enzymes. Although *C. elegans* lacks COX and LOX orthologs, F-series PGs synthesized independently of COX from C20 PUFA precursors have been detected in lipid extracts [20]. *Caenorhabditis elegans* CYP enzymes, including CYP33E2, CYP29A3, and CYP13A12, have been implicated in oxylipin biosynthesis [24–26]. Infective L3 larvae of *A. cantonensis* can take up exogenous arachidonic acid (AA) and incorporate it into phospholipids and neutral lipids, concurrently secreting leukotriene B4—a pro-inflammatory AA metabolite—into the culture medium [27]. While metabolic streamlining that renders parasitic nematodes dependent on host-derived lipids is widely recognized, the enzymes responsible for PUFA and oxylipin biosynthesis, their expression patterns, and the corresponding lipidomic features in parasitic nematodes remain poorly understood.

In this study, we employed lipidomics to characterize the dynamic profiles of PUFAs and oxylipins in L4 larvae residing in the rat brain and in L5 actively migrating to the lungs during the neuro-pulmonary transition. Additionally, we integrated comparative genomics and stage-specific transcriptomics to reconstruct the expression patterns of enzymes involved in PUFA and oxylipin biosynthesis in *A. cantonensis*. These findings provide insights into how the parasitic lifestyle influences PUFA and oxylipin metabolism in nematodes.

## Methods

### Biological resources

Specific pathogen-free Sprague–Dawley rats were purchased from the Medical Experimental Animal Center of Guangdong Province.

### Genome-wide identification of genes for PUFA and oxylipin biosynthesis

The *C. elegans* genome was retrieved from Wormbase ParaSite release WBPS16 [28] and the *A. cantonensis* genome assembly was downloaded from the China National GeneBank Database (CNGBdb) (https://db.cngb.org/). To systematically identify orthologs involved in PUFA and oxylipin biosynthesis, we constructed a reference enzyme set from the Kyoto Encyclopedia of Genes and Genomes (KEGG) pathway database (release 110.0) and employed a multi-tiered search strategy. We first identified orthologous groups between the reference set and both genomes using Orthofinder (version 2.5.2) with default parameters. Concurrently, protein sequences of the reference enzymes were queried against both genomes using tblastn (BLAST+ version 2.6.0) with an e-value threshold of ≤ 1 × 10^–5^. The highest-scoring alignment blocks were merged using Solar (version 0.9.6), and alignment rates below 0.45 were discarded [29]. Then, significant hits were used to define gene models via GeneWise (version 2.2.3), with 500-base pair flanking sequences on both sides to capture complete coding regions [30]. The resulting gene sets produced by Orthofinder and GeneWise were merged, and redundancies were removed. All predicted protein sequences were scanned against the Pfam database (version 28.0) using PfamScan (HMMER version 3.1b2). Only sequences containing the expected catalytic domains were retained as functional candidates; sequences lacking conserved domains were flagged as pseudogenes or gene fragments and excluded from subsequent analyses.

A gene was classified as absent/lost only if it met all of the following criteria: (i) no significant hit in the homology-based search and gene model reconstruction; (ii) no conserved catalytic domain detected by PfamScan; (iii) no expressed sequence corresponding to the reference gene in stage-specific transcriptomes (RNA sequencing reads mapped to the genome using HISAT2, version 2.2.1). To rule out assembly-induced false negatives, we also checked whether the missing gene locus resided near scaffold boundaries or within assembly gaps (> 500 base pairs of ambiguous 'N' regions).

### Transcriptomic analysis

Illumina RNA-seq reads for *C. elegans* (BioProject PRJNA231838) and *A. cantonensis* (BioProject PRJNA350405) were downloaded from the Sequence Read Archive database. Data were analyzed as previously described [31]. FastQC (version 0.11.6) was employed for quality control, and Trim-galore (version 0.6.6) was used for the filtration of low-quality reads. Reads were aligned with Spliced Transcripts Alignment to a Reference (version 2.7.9a) and quantified with RNA-seq by Expectation-Maximization (version 1.3.3) [32, 33]. Fragments per kilobase million values of the genes were visualized as heat maps in TBtools (version 1.098696) [34].

### Preparation of L4-L5 larvae of *A. cantonensis*

Male and female Sprague–Dawley rats, 6–8 weeks old, were inoculated by gavage with ~ 200 infective L3 larvae of *A. cantonensis* isolated from experimentally infected *Biomphalaria glabrata*. L4-L5 larvae were recovered from rat brains and lungs at days post-infection (dpi) 21, 24, 26, and 28, as described [3].

### Oxylipin analysis

For lipid extraction, L4-L5 larvae (≥ 20 mg wet weight) were homogenized with stainless steel beads in 200 μL of extraction solvent using a ball mill. Following bead removal, 20 μL of a 1-μM internal standard mixture was added to each sample. The mixture was vortexed for 10 min and centrifuged at 5000 r.p.m. for 10 min at 4 °C. The extraction was repeated once, and the supernatants were pooled. Oxylipins were then purified using Poly-Sery MAX SPE columns (ANPEL). The eluate was dried under vacuum and reconstituted in 100 μL of methanol/water (1:1, volume/volume) for ultra-performance liquid chromatography-tandem mass spectrometry (LC-MS/MS) analysis. Ultra-performance LC-MS/MS analysis was performed as described previously [35]. Standard solutions (Cayman Chemical) were prepared at 0.01, 0.02, 0.04, 0.1, 0.2, 0.4, 1, 2, 4, 10, 20, 40, 100, 200, and 400 nmol/L, and the corresponding quantitative mass spectrometric peak intensities were recorded for each concentration. Standard curves for individual analytes were generated by plotting the analyte-to-internal standard peak area ratio (*y*-axis) against the corresponding concentration ratio (*x*-axis). Principal component analysis (PCA) was conducted using R prcomp (version 3.5.0). Hierarchical clustering and Pearson correlation analyses were visualized using the pheatmap package (version 1.0.12).

Orthogonal partial-least-squares discriminant analysis (OPLS-DA) was performed on mean-centered data using the OPLSR.Anal function in MetaboAnalystR (version 1.0.1) to partition predictive from orthogonal variation and identify discriminative variables. Differential metabolites were defined by a variable importance in projection score ≥ 1 and |log_2_ fold change|≥ 1. Oxylipins were annotated against the KEGG Compound database and mapped to KEGG pathways. The differential oxylipins in the pathway were analyzed by metabolite set enrichment analysis, and their significance was determined by hypergeometric test *P*-values [35].

### Statistical analysis

Multivariate differences were assessed by PCA and OPLS-DA. Univariate comparisons (PUFAs) were tested by Student’s t-test or one-way ANOVA followed by Tukey’s post hoc test. Correlations were evaluated with Spearman’s and partial correlation coefficients (SPSS 16.0). *P* < 0.05 was considered statistically significant.

## Results

### *A. cantonensis* encodes a streamlined set of PUFA and oxylipin biosynthetic genes compared with free-living *C. elegans*

**Fig. 1:**
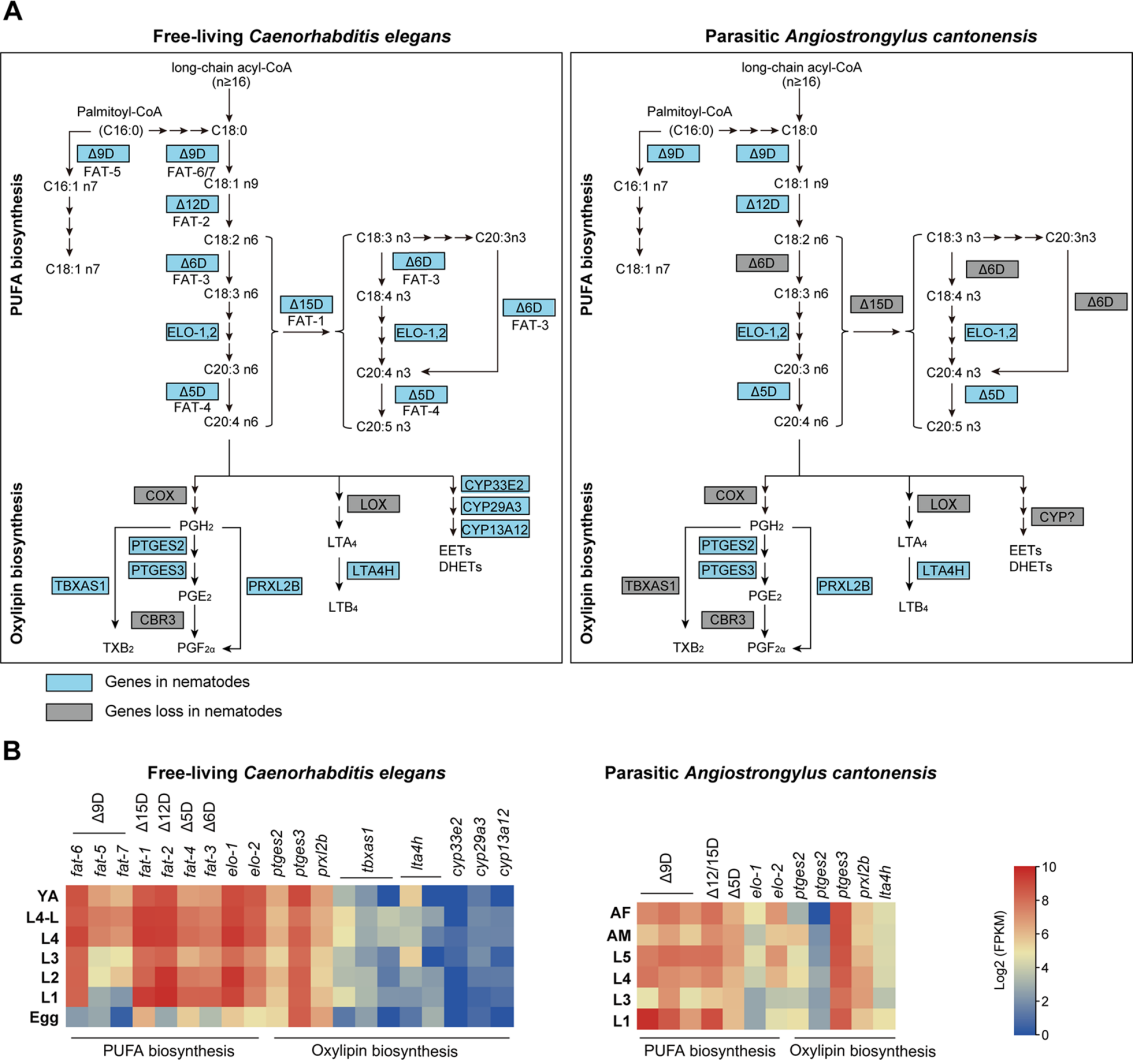
Gene repertoire and expression patterns of PUFA and oxylipin biosynthe tic pathways in *C*. *elegans* and *A*. *cantonensis*. (A) Gene repertoire of PUFA and oxylipin biosynthetic pathways. CYP? means CYP protein that mayexist but has not yet been identified. (B) Expression patterns of enzymes in PUFA and oxylipin biosynthetic pathways. L1-L5: first-five stages of larvae; L4-L: late L4 larvae; YA: young adult; AM: Adult male; AF: Adult female

In *C. elegans*, the canonical *n*-6/*n*-3 pathway is initiated by three successive desaturases. Δ9 desaturase (Δ9D; FAT-5/6/7) converts stearic acid (C18:0) into oleic acid (C18:1, *n*-9), Δ12 desaturase (Δ12D; FAT-2) converts oleic acid into linoleic acid (LA; C18:2, *n*-6). Δ15 desaturase (Δ15D; FAT-1) converts linoleic acid into α-linolenic acid (ALA; C18:3, *n*-3). Subsequent alternating reactions catalyzed by Δ6-desaturase (Δ6D; FAT-3), Δ5-desaturase (Δ5D; FAT-4), and two elongases (*elo-1/elo-2*) extend LA and ALA into *n*-6 and *n*-3 PUFAs. The *C. elegans* genome encodes seven biochemically characterized desaturases (from *fat-1* to *fat-7*) and two elongases (*elo-1* and *elo-2*) (Fig. 1A; Additional file 1: Table S1).

*Angiostrongylus cantonensis* retains only seven putative PUFA biosynthetic genes and has lost the ortholog of Δ6D (Fig. 1A; Additional file 1: Table S1). This reduced set comprises three Δ9-desaturase genes (Δ9D-1, -2, -3), of which Δ9D-2 and Δ9D-3 are 98% identical tandem duplicates on scaffold 97, while Δ9D-1 is more divergent (~ 73% identity to the pair). All three are most closely related to *C. elegans* FAT-6, which catalyzes the conversion of C18:0 to C18:1. *Caenorhabditis elegans* possesses Δ15D (FAT-1) and Δ12D (FAT-2) for the de novo biosynthesis of *n*-6 and *n*-3 PUFAs, the enzymes that are absent in vertebrates [36, 37]. *Angiostrongylus cantonensis* harbors a single ortholog sharing 81.2% and 66.5% identity to *C. elegans* Δ12D and Δ15D, respectively; however, its bifunctional capacity remains to be functionally validated. *Angiostrongylus cantonensis* retains single copies of Δ5D, *elo-1*, and *elo-2* but lacks Δ6D, which is required for the conventional mammalian route to AA and other C20 *n*-6 PUFAs.

Although nematodes naturally lack COX and LOX, *C. elegans* compensates for this via 20 genes that mediate COX/LOX-independent oxylipin synthesis, including *ptges2*, *ptges3*, *prxl2b*, *lta4h*, *tbxas1*, *cyp13a12*, *cyp29a3* and *cyp33e2* (Fig. 1A; Additional file 1: Table S1). *Angiostrongylus cantonensis* has undergone extensive gene loss in this module, retaining only five orthologs: two *ptges2* paralogs, one *ptges3*, one *prxl2b* and a single *lta4h*. Genes corresponding to *cox*, *lox, tbxas1*, *cyp13a12*, *cyp29a3* and *cyp33e2* are absent (Fig. 1A; Additional file 1: Table S1). *Angiostrongylus cantonensis* reveals marked streamlining of both the PUFA and oxylipin biosynthetic pathways, with the reduction being especially severe in oxylipin biosynthesis.

### Transcriptional output of PUFA and oxylipin biosynthetic genes is consistently lower in parasitic *A. cantonensis *than in free-living *C. elegans*

Transcript abundance largely determines enzymatic activity and, consequently, the levels of PUFAs and oxylipins in organisms. To infer the potential enzymatic activities of larvae migrating through host tissues, an analysis was conducted on the stage-specific expression profiles between *A. cantonensis* and *C. elegans*. In *A. cantonensis*, L3 larvae (infective stage) displayed the lowest transcript levels across the life cycle. L1 larvae hatched in rat lungs showed the highest expression of PUFA biosynthetic genes, followed by migrating L4 and L5 larvae. Adult females exhibited stronger expression of these genes than males, a pattern consistent with the greater lipid requirements associated with egg production (Fig. 1B; Additional file 1: Tables S2). In *C. elegans*, eggs exhibited the lowest transcript abundance for both PUFA and oxylipin pathways. Among the three Δ9D paralogs, only *fat-6* was highly expressed in L1-L3 larvae, whereas *fat-5* and *fat-7* were predominantly expressed in L4-L5 larvae, suggesting an increased requirement for unsaturated fatty acids during late larval development (Fig. 1B; Additional file 1: Tables S3).

In *A. cantonensis*, genes encoding Δ9D, Δ12D/Δ15D, Δ5D, and *elo-2* in the PUFA biosynthetic pathway, as well as *ptges3* and *prxl2b* in the oxylipin pathway, showed relatively high transcription levels. Conversely, *elo-1* for PUFA biosynthesis and *ptges2* and *lta4h* for oxylipin biosynthesis were weakly transcribed (Fig. 1B; Additional file 1: Tables S2)*.* In *C. elegans*, desaturases, *elo*, *ptges2, ptges3,* and *prxl2b* showed relatively high expression, whereas *tbxas1*, *lta4h*, *cyp33e2*, *cyp29a3* and *cyp13a12* were weakly transcribed (Fig. 1B; Additional file 1: Table S3). In both species, PUFA biosynthetic genes were transcribed at higher levels than oxylipin biosynthetic genes (Fig. 1B; Additional file 1: Tables S2-S3). However, absolute transcript levels for these genes were substantially lower in *A. cantonensis* than in *C. elegans*, consistent with the observed gene loss. Thus, parasitism in *A. cantonensis* is associated with contraction of the PUFA/oxylipin genetic toolkit and a concomitant downshift in transcriptional activity relative to the free-living *C. elegans*.

### PUFA profiles of *A. cantonensis* during migration from rat brain to lung

LC–MS/MS profiling identified seven PUFAs and 93 oxylipins across L4 and L5 larvae. Total PUFA content in lung-stage L5 larvae reached 7350.6 nmol/g dry weight (DW), surpassing the 5362.6–6863.4 nmol/g DW observed in brain-stage L4-L5 larvae (Table 1; Additional file 1: Table S4). Specifically, *n*-6 PUFA content in lung-stage L5 larvae was 6588.8 nmol/g DW, which was significantly higher than the 4755.0–6129.6 nmol/g DW in brain-stage larvae. In contrast, *n*-3 PUFA levels in lung-stage L5 larvae (761.8 nmol/g DW) did not differ significantly from those in brain-stage larvae (607.6–777.8 nmol/g DW) (Table 1; Additional file 1: Table S4). These findings indicate stage- and tissue-specific accumulation of *n*-6 PUFAs during pulmonary migration.

**Table 1:** Concentrations of polyunsaturated fatty acids (*PUFAs*) in *Angiostrongylus cantonensis* from rats (nmol/g dry weight, *n* = 5)

Fatty acids	Fourth- to fifth-stage (L4-L5) larvae in rat brain				L5 in rat lung
	R21B (L4)	R24B (L4-L5)	R26B (L4-L5)	R28B (L5)	R28L (L5)
C18:2 (*n*-6)	2306.0 ± 310.9	2544.0 ± 239.5	2332.0 ± 58.1*^b^	2000.0 ± 343.6*^b^	2740.0 ± 490.0*^d^
C18:3 (*n*-6)	218.4 ± 24.6	246.0 ± 23.9	232.4 ± 27.1	206.0 ± 50.7	330.6 ± 61.0*^a^*^b^*^c^*^d^
C18:3 (*n*-3)	74.2 ± 15.0	77.8 ± 10.5	67.9 ± 13.3	58.8 ± 18.8	143.8 ± 56.7*^a^*^b^*^c^*^d^
C20:3 (*n*-6)	502.6 ± 61.6	629.6 ± 53.3*^a^	616.8 ± 69.2*^a^	517.0 ± 114.6*^b^	610.2 ± 101.4*^a^
C20:4 (*n*-6)	2428.0 ± 358.6	2710.0 ± 299.3	2332.0 ± 81.7*^b^	2032.0 ± 468.7*^b^	2908.0 ± 470.2*^c^*^d^
C20:5 (*n*-3)	355.8 ± 38.9	366.6 ± 55.0	345.4 ± 44.1	280.0 ± 47.7*^a^*^b^*^c^	252.2 ± 30.2*^a^*^b^*^c^
C22:6 (*n*-3)	347.8 ± 35.2	289.4 ± 30.3*^a^	266.6 ± 29.9*^a^	268.8 ± 88.9	365.8 ± 65.8*^c^
*n*-6 PUFAs	5455.0 ± 745.9	6129.6 ± 554.4	5513.2 ± 139.1*^b^	4755.0 ± 957.6*^b^	6588.8 ± 1107.3*^c^*^d^
*n*-3 PUFAs	777.8 ± 83.1	733.8 ± 86.4	679.9 ± 78.9*^a^	607.6 ± 131.5*^a^	761.8 ± 113.0
*n*-6/*n*-3 PUFAs	7.0	8.4	8.1	7.8	8.7

^a^Compared with R21B

^b^Compared with R24B

^c^Compared with R26B

^d^Compared with R28B

* P < 0.05

LC–MS/MS analysis further revealed dynamic shifts in individual fatty acids. Relative to brain-resident larvae at 24–26 dpi, brain-stage L5 larvae at 28 dpi exhibited modest decreases in C18:2 (*n*-6), C20:3 (*n*-6), C20:4 (*n*-6), and C20:5 (*n*-3), whereas C22:6 (*n*-3) remained stable (Table 1; Additional file 1: Table S4). In contrast, lung-stage L5 larvae displayed significant increases in C18:2 (*n*-6), C18:3 (*n*-6), C18:3 (*n*-3), C20:4 (*n*-6), and C22:6 (*n*-3), concomitant with decreases in C20:5 (*n*-3) (Table 1; Additional file 1: Table S4). These data demonstrate progressive PUFA accumulation during nematode development and tissue migration.

### Oxylipin profiles of *A. cantonensis* during migration from rat brain to lung

**Fig. 2A–C:**
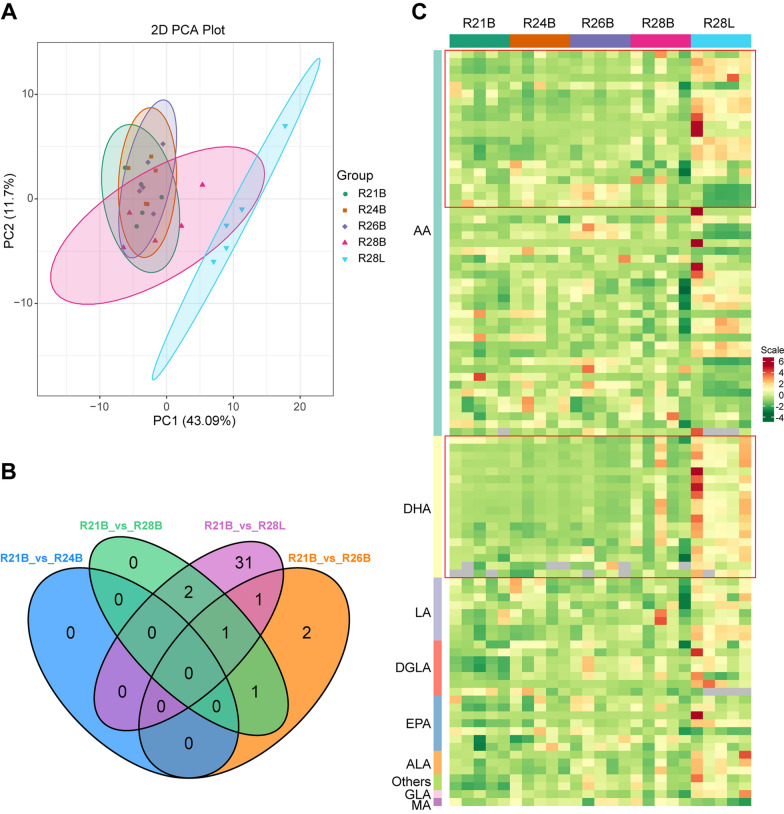
Oxylipin profiles of *Angiostrongylus cantonensis* L4-L5 larvae during rat cerebral-to-pulmonary migration. **A** Principal component analysis (PCA) score plot of oxylipin profiles reveals the separation between cerebral and pulmonary larvae. PC1 and PC2 explain 43.09% and 11.70% of the total variance, respectively. **B** Venn diagram of differentially abundant oxylipins. **C** Hierarchical clustering of oxylipin levels. The scale indicates the level after standardized treatment (deep red = high). Metabolites with significant inter-group differences are shown within the red frames. L4 and L5 larvae collected from the rat brain at days post-infection (dpi) 21 (*R21B*), 24 (*R24B*), 26 (*R26B*), and 28 (*R28B*); L5 larvae collected from the rat lung at dpi 28 (*R28L*).* AA* Arachidonic acid C20:4 (*n*-6);* DHA* docosahexaenoic acid C22:6 (*n-*3);* LA* linoleic acid C18:2 (*n-*6);* DGLA* dihomo-γ-linolenic acid C20:3 (*n-*6);* EPA* eicosapentaenoic acid C20:5 (*n-*3);* ALA* α-linolenic acid C18:3 (*n-*3);* GLA* γ-linolenic acid C18:3 (*n-*6);* MA* mead acid C20:3 (*n-*9)

PCA of LC–MS/MS lipidomics revealed clear segregation between brain-resident L4-L5 larvae and lung-migrating L5 larvae (Fig. 2A). Total oxylipin content in brain-stage larvae ranged from 18.9 to 20.9 nmol/g DW, whereas lung-stage larvae exhibited a near-doubling to 35.6 nmol/g DW (Additional file 1: Table S4). In brain-stage larvae, 5,6-epoxyeicosatrienoic acid (5,6-EET) was the most abundant oxylipin (3.5–5.7 nmol/g DW), with all other oxylipins present at < 1.0 nmol/g DW (Additional file 1: Table S4). In contrast, lung-stage L5 larvae were enriched in 12-HETE (5.4 nmol/g DW), followed by 5,6-EET, 5-HETE, TXB_2_, 20-hydroxy docosahexaenoic acid (20-HDHA), 17-HDHA, and 14-HDHA, which all exceeded 1.0 nmol/g DW (Additional file 1: Table S4).

**Fig. 3:**
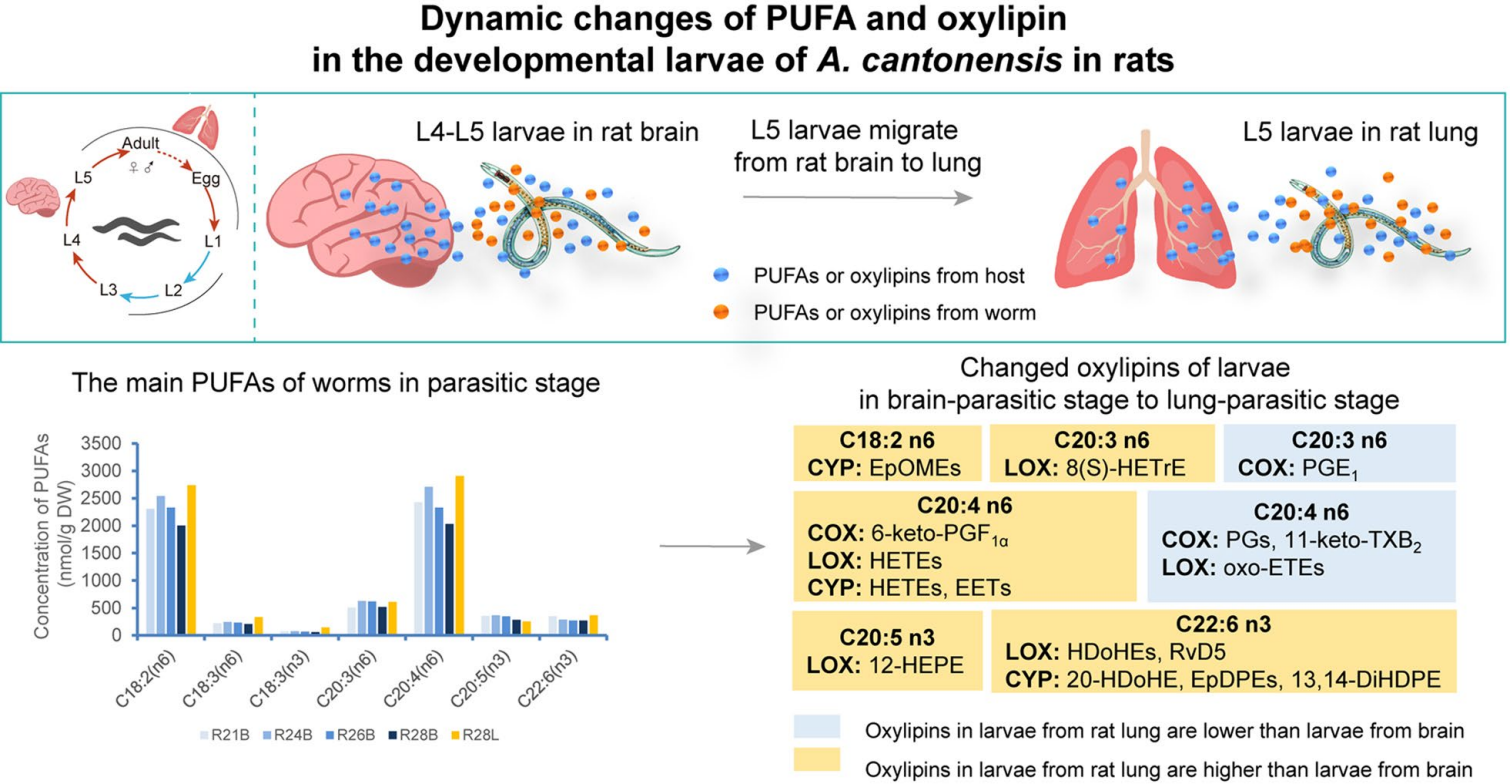
PUFA and oxylipin dynamics in *Angiostrongylus cantonensis* L4-L5 larvae during rat cerebral-to-pulmonary migration

Differential oxylipin analysis revealed from zero to five significantly altered oxylipins in brain-stage larvae versus 12–37 in lung-stage larvae (Fig. 2B; Additional file 2: Table S5). Notably, tetranor-12(S)-HETE, 12-HETE, 12-hydroxyeicosapentaenoic acid (12-HEPE), and 14(S)-hydroxydocosahexaenoic acid [14(S)-HDoHE] were elevated by ~ 10.0-, 12.0-, 8.6-, and 10.0-fold, respectively, in lung-stage larvae compared with brain-stage larvae (Additional file 1: Table S4). Clustering analysis indicated that these differential oxylipins were predominantly downstream metabolites of AA, DHA, DGLA, LA, and EPA—especially AA and DHA—generated via COX, LOX, and CYP pathways (Fig. 2C; Additional file 1: Table S4; Additional file 2: Table S6). Except for some PGs, 11-keto-TXB_2_, and oxo-ETEs derived from DGLA or AA, most oxylipins synthesized from LA, AA, EPA, and DHA were more abundant in lung-stage larvae (Fig. 2C; Fig. 3; Additional file 1: Tables S4, S6). This pattern aligns with the elevated precursor levels (LA, AA, DHA) detected by LC–MS/MS in lung-stage L5 larvae (Figs. 2C and 3; Additional file 1: Table S4; Additional file 2: Table S6), suggesting that substrate availability drives oxylipin amplification during pulmonary migration.

## Discussion

PUFAs and their oxylipins regulate diverse physiological and pathological processes in nematodes. In *C. elegans*, RNA interference-mediated knockdown of *fat-2*, *fat-6*, *fat-7* or *elo-2* reduces fatty acid pools, shortens the lifespan, and disrupts development [38]. Our findings indicate that *A. cantonensis* retains a contracted PUFA/oxylipin toolkit: its genome lacks Δ6D, *cox*, *lox*, *tbxas1*, *cyp13a12*, *cyp29a3*, and *cyp33e2*. Specifically, all three *A. cantonensis* Δ9 desaturases cluster with *C. elegans* FAT-6 (C18:0 to C18:1) rather than FAT-5, and the single Δ12/15-desaturase is phylogenetically closer to FAT-2 (Δ12) than to FAT-1 (Δ15). This pattern of metabolic streamlining is consistent with the broader evolutionary trajectory of parasitic nematodes [39].

*Angiostrongylus cantonensis* larvae were abundant in PUFAs, with *n*-6 PUFAs reaching their highest levels in lung-stage L5 larvae, consistent with the relatively high transcription of Δ9D, Δ12D, Δ5D, and *elo-2* observed in this stage. The distinct lipid profiles of brain-resident and lung-migrating larvae are probably shaped, at least in part, by the differing physiological environments of these organs. The brain is protected by the blood–brain barrier, which restricts the passage of large molecules, immune cells, and plasma lipids, potentially creating a relatively sequestered and lipid-limited niche. In contrast, the pulmonary vasculature is highly permeable, richly supplied with circulating fatty acids, and characterized by active immune cell trafficking and AA release during inflammation. These differences may explain why lung-stage L5 larvae exhibit higher total PUFA and *n*-6 PUFA levels than brain-stage larvae. Quantitative lipidomic analyses of *Ascaris suum* have revealed that its developmental lipid profile closely mirrors the changing host microenvironment [40]. Comparable stage-specific PUFA expansion has been observed in *Dictyocaulus viviparus*, whose parasitic stages accumulate a broader PUFA repertoire than free-living larvae, presumably reflecting host tissue availability [41]. Whether *A. cantonensis* actively scavenges host lipids (e.g., through hematophagy) or passively accumulates them from the surrounding milieu cannot be resolved with the current data.

Oxylipin levels were also markedly higher in lung-stage than in brain-stage larvae (35.6 nmol/g DW versus ≤ 20.9 nmol/g DW). Lung-stage larvae were enriched in AA- or DHA-derived 6-keto-PGF_1α_, HETEs, EETs, and HDoHEs, as well as epoxy docosapentaenoic acids, yet contained fewer COX-derived PGs and 11-keto-TXB_2_, as well as lower levels of CYP-mediated oxo-ETEs. In mammals, 12-HETE can act as a pro-inflammatory chemoattractant for eosinophils [42], whereas 17-HDoHE and 16,17-epoxy docosapentaenoic acid exert anti-hyperalgesic and pro-resolving effects [42]. EETs antagonize NF-κB signaling and leukocyte adhesion [43], while 5-oxo-ETE provokes oxidative bursts and degranulation in cytokine-primed cells [44]. The observed shift toward anti-inflammatory LOX/CYP metabolites and away from pro-inflammatory COX products may reflect an immunomodulated environment that tolerates the rapid growth and onset of oviposition in L5 larvae. However, it remains equally plausible that these changes simply mirror the baseline oxylipin composition of rat lung tissue rather than represent parasite-driven immunomodulation.

A critical ambiguity remains regarding the biosynthetic origin of the detected oxylipins. Our genomic and transcriptomic analyses indicate the absence of canonical COX, LOX, and CYP orthologs, as well as Δ6 desaturase, alongside transcriptional downregulation of residual *ptges2* and *lta4h*. These findings are compatible with the hypothesis that *A. cantonensis* acquires oxylipins (or their precursors) from the host. Nevertheless, we cannot formally exclude three alternative explanations. First, the oxylipins may represent host-derived metabolites that adsorbed to the cuticle or contaminated the parasite preparation during isolation from rat brain or lung tissue. Although we took care to wash parasites extensively, trace host tissue or blood cannot be completely ruled out, given the small sample sizes and the intimate contact between larvae and host tissue. Second, *A. cantonensis* may possess non-canonical or highly divergent enzymes capable of synthesizing oxylipins despite the absence of obvious *C. elegans* orthologs. The possibility of cryptic biosynthetic routes remains open until enzymatic assays or heterologous expression studies are performed. Third, host enzymes may convert scavenged parasite-released PUFAs into oxylipins within the local microenvironment, which are then detected in the worm extracts. These scenarios are not mutually exclusive, and the present data do not allow us to discriminate between them. Consequently, any inference that *A. cantonensis* “predominantly” scavenges host oxylipins should be regarded as provisional.

These observations contribute a preliminary developmental lipidomic profile for *A. cantonensis* and implicate tissue-specific oxylipin signatures as potential correlates of parasite–host interaction. Comparative genomic analyses across additional nematode lineages, coupled with functional validation of the residual Δ12/15-desaturase and *ptges2*/*lta4h* orthologs, will be essential to resolve questions of oxylipin provenance and evolutionary loss. Although our data clearly demonstrate stage-specific lipidomic remodeling during neuro-pulmonary migration, the relative contributions of parasite biosynthesis, host scavenging, and environmental contamination to the observed oxylipin pool remain to be experimentally dissected.

## Conclusions

Parasitism has trimmed both the gene repertoire and transcriptional output of PUFA/oxylipin biosynthesis in *A. cantonensis*. The stage-specific lipidomic remodeling observed during neuro-pulmonary migration correlates with the distinct microenvironments of the rat brain and lung, though this association does not establish active exploitation of host immunity. These findings provide a foundation for understanding the evolutionary adaptation of lipid metabolism in parasitic nematodes, pending functional studies to clarify oxylipin provenance.

## Supplementary Information

Additional file 1: Tables S1–S4. Table S1. Genes in* Caenorhabditis elegans* and* Angiostrongylus cantonensis* for PUFA and oxylipin biosynthesis. Table S2. Expression pattern of genes in PUFA and oxylipin biosynthesis in* Angiostrongylus cantonensis*. Table S3. Expression pattern of genes in PUFA and oxylipin biosynthesis in* Caenorhabditis elegans*. Table S4. The contents of PUFAs and oxylipins in the L4-L5 larvae of* Angiostrongylus cantonensis* from rat brain and lung.

Additional file 2: Tables S5 and S6. Table S5. OPLS-DA analysis of differential oxylipins in* Angiostrongylus cantonensis* at dpi 21, 24, 26 and 28 from rat brain and dpi 28 from rat lung. Table S6. Differential oxylipins in the L4-L5 larvae of* Angiostrongylus cantonensis* based on OPLS-DA analysis.

## Data Availability

The datasets analyzed during the current study are available from public databases (Wormbase, CNGBdb, and The National Center for Biotechnology Information). All relevant accession numbers of genomes and transcriptomes are listed in Additional file 1: Table S1-S3.
