# Supplementary material for: Polyunsaturated fatty acid and oxylipin profiles of Angiostrongylus cantonensis during cerebral-to-pulmonary migration illuminate parasite and host microenvironmental lipid signatures
Source: Parasit Vectors. 2026 Jun 2;19:323. doi: 10.1186/s13071-026-07469-z (PMC13455368; doi:10.1186/s13071-026-07469-z)
Supplement: Supplementary file 1 [file 13071_2026_7469_MOESM2_ESM.docx]

**Supplementary Tables:**

**Table S5 OPLS-DA analysis of differential oxylipins in *A. cantonensis* at dpi 21, 24, 26 and 28 from rat brain and dpi 28 from rat lung (n= 5)**

| Groups | Total number | Decreased number | Increased number | variance explained |
| --- | --- | --- | --- | --- |
| R21B vs R24B | 0 | 0 | 0 | **R²X = 0.22, R²Y = 0.94, Q² = 0.67** |
| R21B vs R26B | 5 | 1 | 4 | **R²X = 0.28, R²Y = 0.94, Q² = 0.77** |
| R21B vs R28B | 4 | 3 | 1 | **R²X = 0.20, R²Y = 0.78, Q² = 0.56** |
| R21B vs R28L | 35 | 10 | 25 | **R²X = 0.53, R²Y = 0.94, Q² = 0.92** |
| R24B vs R26B | 1 | 1 | 0 | **R²X = 0.19, R²Y = 0.84, Q² = 0.58** |
| R24B vs R28B | 0 | 0 | 0 | **R²X = 0.21, R²Y = 0.76, Q² = 0.40** |
| R24B vs R28L | 37 | 11 | 26 | **R²X = 0.54, R²Y = 0.97, Q² = 0.94** |
| R26B vs R28B | 0 | 0 | 0 | **R²X = 0.15, R²Y = 0.82, Q² = 0.21** |
| R26B vs R28L | 27 | 9 | 18 | **R²X = 0.52, R²Y = 0.98, Q² = 0.95** |
| R28B vs R28L | 12 | 6 | 6 | **R²X = 0.40, R²Y = 0.84, Q² = 0.77** |

Note: R21B, R24B, R26B, R28B, R28L: L4 and L5 larvae collected from rat brain at dpi 21, 24, 26, 28 and L5 larvae from rat lung at dpi 28.

**Table S6 Differential oxylipins in the L4-L5 larvae of *A. cantonensis* (n=5, nmol/g Dry weight) based on OPLS-DA analysis**

| Feature | PUFAs | Pathway | Oxylipins | | | |
| --- | --- | --- | --- | --- | --- | --- |
|  |  |  | R21B vs R28L | R24B vs R28L | R26B vs R28L | R28B vs R28L |
| Increased | C20:4 n6 | COX | 6-keto-PGF_1α_ | 6-keto-PGF_1α_ | 6-keto-PGF_1α_ | 6-keto-PGF_1α_ |
|  |  | LOX | 5-HETE, 8-HETE, 9-HETE, 12-HETE, tetranor-12(S)-HETE, 15-HETE, 5(S),15(S)-DiHETE | 8-HETE, 9-HETE, 11(S)-HETE, 12-HETE, tetranor-12(S)-HETE | 8-HETE, 12-HETE, tetranor-12(S)-HETE, 5(S),15(S)-DiHETE | 12-HETE, tetranor-12(S)-HETE |
|  |  | CYP | 16-HETE, 5,6-DiHETrE, 8,9-EET, 11,12-EET, 14,15-EET | 16-HETE, 17-HETE, 8,9-EET, 11,12-EET, 14,15-EET | 17-HETE, 5,6-DiHETrE | -- |
|  | C22:6 n3 | LOX | 4-HDoHE, 8-HDoHE, 10-HDoHE, 11-HDoHE, 16-HDoHE, 17-HDoHE | 4-HDoHE, 8-HDoHE, 10-HDoHE, 11-HDoHE, 13-HDoHE, 14(S)-HDoHE, 16-HDoHE, 17-HDoHE, RvD5 | 4-HDoHE, 7-HDoHE, 8-HDoHE, 10-HDoHE, 11-HDoHE, 13-HDoHE, 14(S)-HDoHE, 16-HDoHE, 17-HDoHE | 10-HDoHE, 13-HDoHE, 14(S)-HDoHE, 16-HDoHE, 17-HDoHE |
|  |  | CYP | 16,17-EpDPE, 19,20-EpDPE | 20-HDoHE, 16,17-EpDPE, 19,20-EpDPE | 20-HDoHE, 16,17-EpDPE, 19,20-EpDPE | 13,14-DiHDPE |
|  | C20:3 n6 | LOX | 8(S)-HETrE | -- | -- | -- |
|  | C18:2 n6 | CYP | 9,10-EpOME, 12,13-EpOME | 9,10-EpOME, 12,13-EpOME | -- | -- |
|  | C20:5 n3 | LOX |  |  | 12-HEPE | 12-HEPE |
| Decreased | C20:4 n6 | COX | PGE_2_, PGD_2_, PGA_2_, PGB_2_, 15-keto-PGE_2_, 13,14-dihydro-15-keto PGE_2_, 11β-PGE_2_, 11-keto-TXB_2_ | PGE_2_, PGD_2_, PGA_2_, PGB_2_, 15-keto-PGE_2_, 13,14-dihydro-15-keto PGD_2_, 11β-PGE_2_, 11-keto-TXB_2_ | PGE_2_, PGD_2_, PGA_2_, PGB_2_, PGJ_2_, 13,14-dihydro-15-keto PGD_2_, 13,14-dihydro-15-keto PGE_2_, 11β-PGE_2_ | PGE_2_, PGD_2_, PGA_2_, PGB_2_, 13,14-dihydro-15-keto PGD_2_, 11β-PGE_2_ |
|  |  | LOX | 5-oxo-ETE, 15-oxo-ETE | 5-oxo-ETE, 15-oxo-ETE | -- | -- |
|  | C20:3 n6 | COX | PGE_1_ | PGE_1_ | -- | -- |

Note: -- means no difference was found. R21B, R24B, R26B, R28B, R28L: L4 and L5 larvae collected from rat brain at dpi 21, 24, 26, 28 and L5 larvae from rat lung at dpi 28.
